# Case Report: Takotsubo Cardiomyopathy in Bickerstaff Brainstem Encephalitis Triggered by COVID-19

**DOI:** 10.3389/fneur.2021.822247

**Published:** 2021-12-24

**Authors:** Mizuki Kimura, Shunta Hashiguchi, Kenichi Tanaka, Manato Hagiwara, Keita Takahashi, Yosuke Miyaji, Hideto Joki, Hiroshi Doi, Michiaki Koga, Hideyuki Takeuchi, Fumiaki Tanaka

**Affiliations:** ^1^Department of Neurology and Stroke Medicine, Yokohama City University Graduate School of Medicine, Yokohama, Japan; ^2^Department of Neurology and Clinical Neuroscience, Yamaguchi University Graduate School of Medicine, Ube, Japan

**Keywords:** Bickerstaff brainstem encephalitis, anti-GQ1b ganglioside antibody, Takotsubo cardiomyopathy, intravenous immunoglobulin therapy, coronavirus disease 2019, transthoracic echocardiogram, hemodynamic instability

## Abstract

Takotsubo cardiomyopathy (TCM) is a stress-induced cardiomyopathy triggered by critical illness including severe neurological disorders. However, an association between TCM and Bickerstaff brainstem encephalitis (BBE) has rarely been described. During the current coronavirus disease 2019 (COVID-19) pandemic, growing evidence indicates that COVID-19 often leads to various neurological disorders, but there are few reports of an association between COVID-19 and BBE. Here we report a case of TCM associated with BBE triggered by COVID-19, which subsided with immunotherapy for BBE. Both transthoracic echocardiography and electrocardiography led to early and accurate diagnosis of TCM. Sustained hemodynamic instability due to TCM was immediately lessened with immunotherapy whereas additional plasmapheresis and immunotherapy were required to treat BBE. This case indicates that BBE might follow COVID-19 and TCM should be considered when hemodynamic status remains unstable in a patient with BBE.

## Introduction

Coronavirus disease 2019 (COVID-19), which is caused by severe acute respiratory syndrome coronavirus-2 (SARS-CoV-2), often leads to various neurological disorders such as ischemic stroke, encephalomyelitis, necrotizing myositis, and Guillain-Barré syndrome (GBS) (1–3). A proposed mechanism for the neurological complications of COVID-19 is direct SARS-CoV-2 invasion of neural cells such as neurons, glial cells, and vascular endothelial cells through angiotensin converting enzyme 2 receptor (4). The mechanism of neurological autoimmunity associated with COVID-19 remains uncertain.

Bickerstaff brainstem encephalitis (BBE) is a rare autoimmune encephalitis caused by autoantibodies against gangliosides such as GQ1b, present in ~75% of patients with BBE (5, 6). BBE involves acute onset of bilateral ophthalmoparesis, ataxia, impaired consciousness, and pyramidal signs. The prognosis is usually good (7). Although GBS often follows COVID-19 (1–3), there are few reports of BBE triggered by COVID-19 (8).

Takotsubo cardiomyopathy (TCM) is a stress-induced cardiomyopathy that presents with malignant arrhythmias and hemodynamic instability. There are specific myocardial wall motion abnormalities and elevated levels of myocardial enzymes. It is occasionally fatal (9). TCM is triggered by critical physiological and psychological events that are hypothesized to be associated with excessive catecholamine release (10), such as intense fear, pain, anxiety; surgery; natural disasters; and infectious diseases including COVID-19 (11). Moreover, severe neurological disorders such as subarachnoid hemorrhage, intracerebral hemorrhage, stroke, epilepsy, migraine, encephalitis, traumatic brain injury, and amyotrophic lateral sclerosis are also associated with TCM (12). To date, an association between TCM and BBE has rarely been described, although GBS is often accompanied by TCM (13).

Here we report a rare case of TCM associated with BBE triggered by COVID-19, which subsided with immunotherapy for BBE. Like GBS, BBE might follow COVID-19 and an association with TCM should be considered when there is sustained hemodynamic instability with BBE.

## Case Report

**Figure 1:**
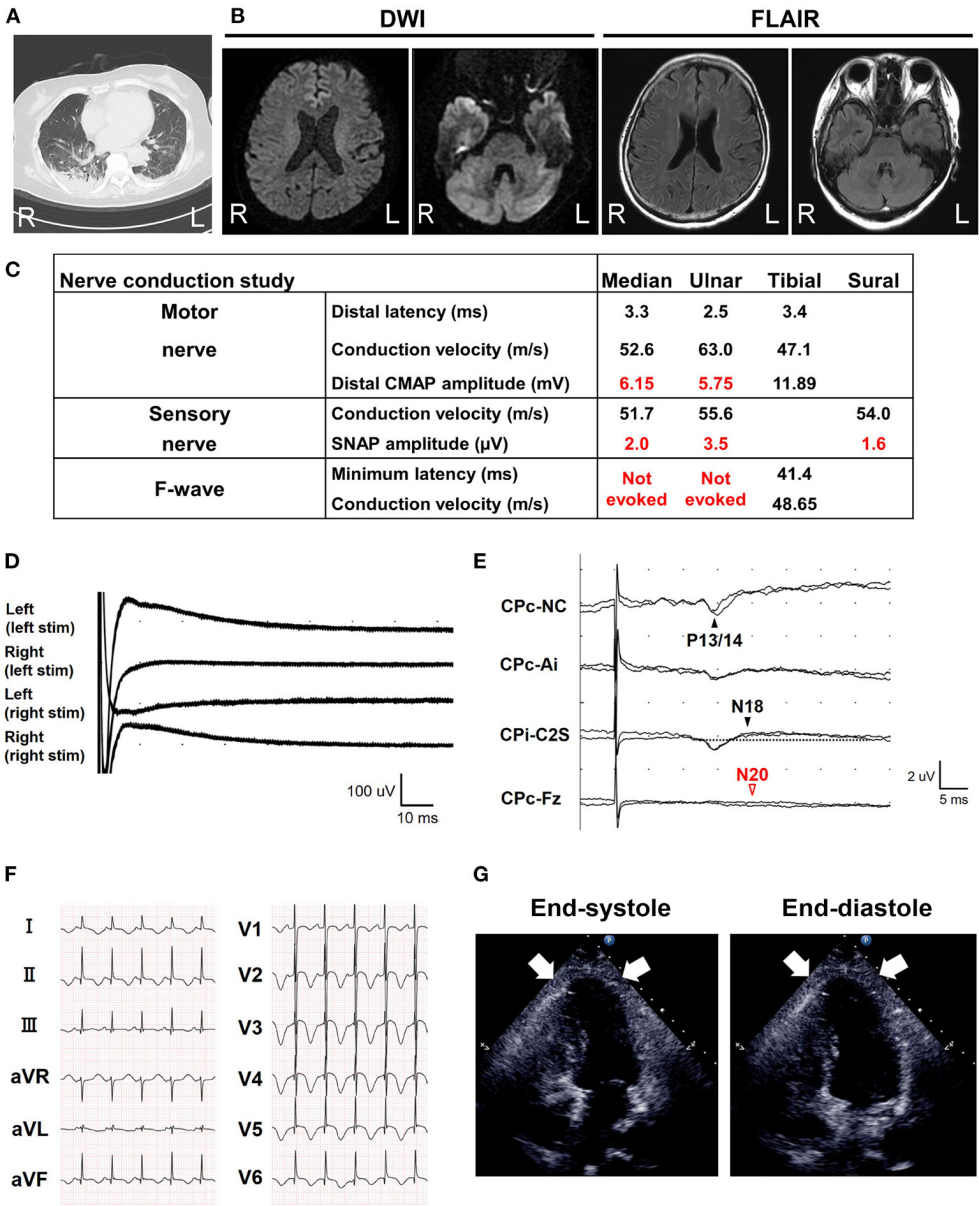
Brain magnetic resonance imaging (MRI) and electrophysiological tests. **(A)** Chest computed tomography detected possible COVID-19 pneumonia on admission. **(B)** Axial brain MRI diffusion weighted imaging (DWI) and fluid-attenuated inversion recovery (FLAIR) images on admission. No abnormalities were detected. **(C)** Nerve conduction study on the 6th day from onset (i.e., hospital day 2). The motor nerve conduction study revealed a slight reduction in compound muscle action potential amplitudes and no conduction block with an apparent increase of stimulation threshold. The orthodromic sensory nerve conduction study showed a reduction in sensory nerve action potentials. F-wave examination exhibited no responses in the upper and lower extremities. CMAP, compound muscle action potential; SNAP, sensory nerve action potential. **(D)** Blink reflex test on day 7 (i.e., hospital day 3) showed bilateral loss of both R1 and R2 with bilateral trigeminal nerve stimulation. **(E)** Median nerve somatosensory evoked potential testing on day 6 (i.e., hospital day 2). P13/14 and N18 were normally evoked, whereas N20 was lost. These results suggested interruption of the somatosensory pathway in the brainstem. Ai, the ipsilateral earlobe. CPc and CPi, the centroparietal electrode contralateral and ipsilateral to the stimulation, respectively. C2S, the spinous process over the second cervical spine. Fz, the midline frontal electrode. NC, non-cephalic reference on the contralateral shoulder. **(F)** Standard 12-lead electrocardiography on admission (day 5) demonstrated inverted T-waves in leads I, II, aVF, and V1–V6. **(G)** Transthoracic echocardiography on admission (day 5). Images in end-diastole (left) and end-systole (right) revealed segmental wall motion abnormalities with apical akinesis (arrows) and hyperkinesis in the basal segments, which was compatible with Takotsubo cardiomyopathy.

**Figure 2:**
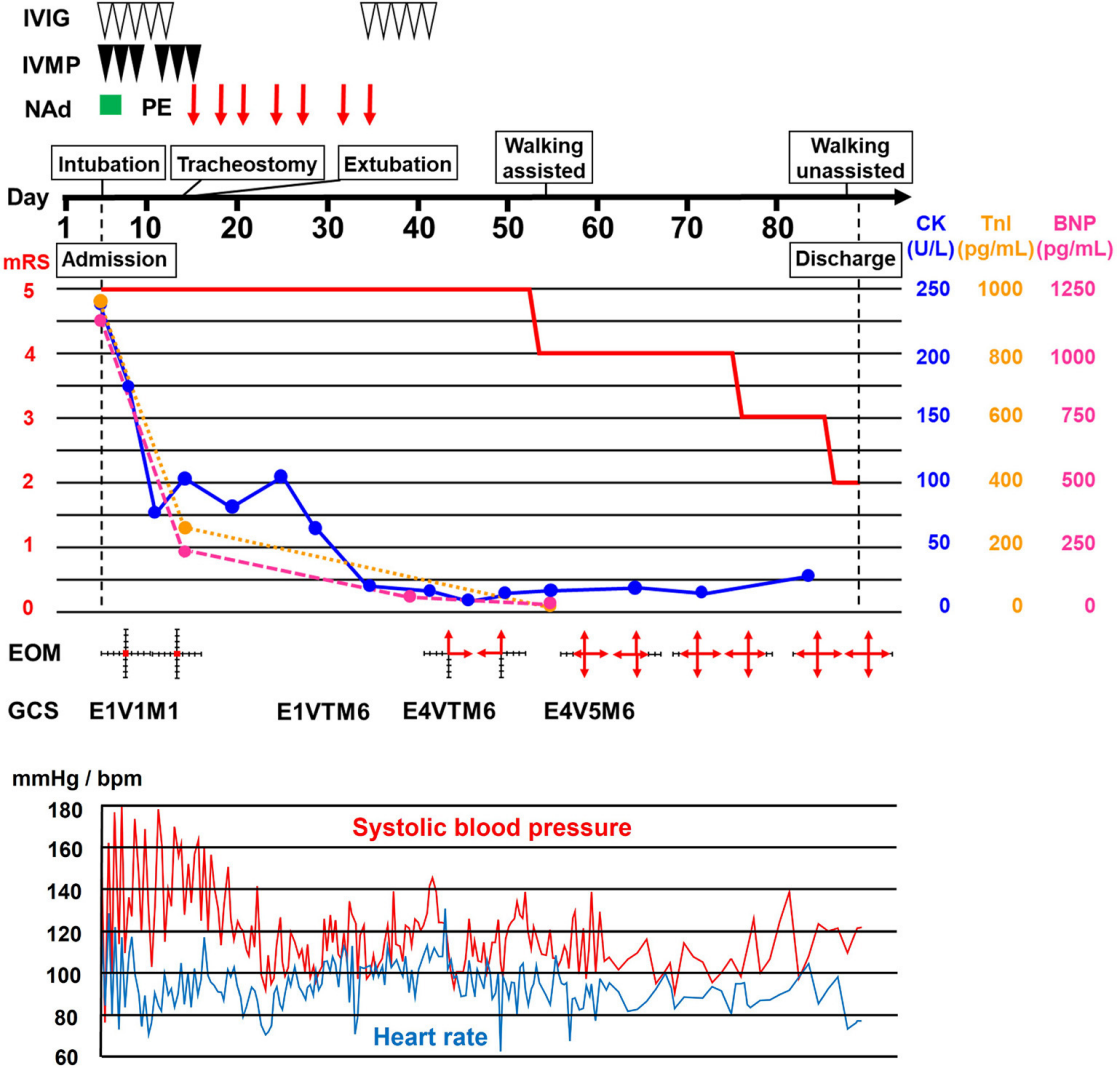
Clinical course. Clinical and treatment course. The horizontal axis represents the day from onset. BNP, B-type natriuretic peptide; CK, creatine kinase; EOM, extraocular movement; GCS, Glasgow Coma Scale; IVIG, intravenous immunoglobulin therapy (400 mg/kg/day for 5 consecutive days); IVMP, intravenous methylprednisolone pulse therapy (1 g/day for 3 consecutive days); mRS, modified Rankin scale (0, no symptoms; 1, no significant disability; 2, slight disability, able to look after own affairs without assistance, but unable to carry out all previous activities; 3, moderate disability, require some help, but able to walk unassisted; 4, moderately severe disability, unable to attend to own bodily needs without assistance, and unable to walk unassisted; 5, severe disability, require constant nursing care and attention, bedridden, incontinent); NAd, noradrenaline administration; PE, plasma exchange; TnI, troponin I.

A 68-year-old Japanese woman was admitted to another hospital due to acute onset of dysarthria and gait disturbance (day 1), after antecedent symptoms of upper respiratory tract infection such as fever and cough lasting 2 weeks. She had not received any COVID-19 vaccination before onset. She developed altered mental status and rapidly deteriorated within 2 days. She was transferred to our hospital on day 5. She had a history of hypertension, but family history was unremarkable. On admission, she was bed-ridden and unconscious [modified Rankin Scale (mRS), grade 5; Glasgow Coma Scale (GCS), E1V1M1] with blood pressure of 152/114 mmHg, heart rate of 120 beats per minute, and body temperature of 33.2°C. Chest computed tomography (CT) detected bilateral pneumonia, presumably caused by COVID-19 (Figure 1A). Although her polymerase chain reaction results for SARS-CoV-2 in sputum and cerebrospinal fluid were negative, she was seropositive for anti–SARS-CoV-2 antibodies (Elecsys Anti–SARS-CoV-2, Roche Diagnostics), which was evidence of SARS-CoV-2 infection. Because of airway obstruction secondary to glossoptosis, she was intubated and supported by mechanical ventilation. Her eyes were fixed in position and she had complete flaccid paralysis with diminished tendon reflexes in all extremities. No pathological reflex was observed in all extremities. There was mydriasis (pupils were 6 mm in diameter bilaterally) and no pupillary light responses accompanied by lack of oculocephalic reflex, gag reflex, corneal reflex, and jaw jerk. Cerebrospinal fluid analysis revealed normal cell count (2 cells/μL) and protein level (20 mg/dL) with high glucose level (164 mg/dL) and the presence of oligoclonal bands. Brain magnetic resonance imaging (MRI) detected no significant abnormalities (Figure 1B). Electroencephalography demonstrated no neither evidence of seizure activity nor response to photic and sound stimuli. A nerve conduction study revealed a slight reduction in both compound muscle action potentials and sensory nerve action potentials. F-wave examination showed no responses in the upper and lower extremities (Figure 1C). Auditory brainstem responses were completely normal, although there was bilateral loss of both R1 and R2 in the blink reflex. During median nerve somatosensory evoked potential testing, normal P13/14 and N18 were detected, whereas N20 was lost (Figures 1D,E). The results of these neurophysiological studies suggest the presence of mild motor and sensory axonal neuropathy and brainstem dysfunction. Autoantibodies against gangliosides such as GQ1b, GT1a, and GM1/GT1a complex were positive in the serum. There was no serological evidence of recent infection with *Campylobacter jejuni, Haemophilus influenzae, Mycoplasma pneumoniae*, cytomegalovirus, or Epstein-Barr virus. Accordingly, she was diagnosed as having probable BBE (5) triggered by COVID-19. On the other hand, electrocardiography showed deep T-wave inversions in multiple leads (Figure 1F). Laboratory testing detected elevated levels of troponin I (967.1 pg/mL), creatine kinase (CK) (287 mg/mL), and B-type natriuretic peptide (BNP) (1,110.2 pg/mL). CT angiography revealed no significant coronary stenosis. Transthoracic echocardiography showed apical akinesis with preserved basal function and a depressed ejection fraction of ~50%, consistent with the diagnosis of TCM (Figure 1G). Both inotropic support and heparinization were introduced promptly for the management of TCM. For BBE, two cycles of intravenous methylprednisolone pulse therapy (IVMP, 1 g/day for 3 consecutive days) and intravenous immunoglobulin therapy (IVIG, 400 mg/kg/day for 5 consecutive days) were simultaneously started on day 6 (i.e., hospital day 2) because sustained hemodynamic instability did not allow for plasmapheresis (Figure 2). Following the first immunotherapies, her hemodynamic status stabilized and levels of CK, troponin I, and BNP decreased, but neurological status did not improve. Next, plasma exchange (PE) was performed 7 times, starting on day 14. PE immediately enabled extubation. She became able to obey some commands although it was hard to open her eyes actively because of external ophthalmoplegia (GCS, E1VTM6) on day 24 (Figure 2). Therefore, additional IVIG was required to achieve a gradual but substantial neurological recovery. Follow-up echocardiography on day 66 showed a marked decrease in apical ballooning of the left ventricle, indicating good recovery from TCM. Finally, she became able to walk without assistance (mRS, grade 2) on day 87 (i.e., hospital day 83). At discharge, she had residual double vision and bilateral disturbance in abduction.

## Discussion

BBE is an autoimmune neurological disorder affecting both the central and peripheral nervous systems that is usually associated with antibodies against gangliosides such as GQ1b. It is clinically characterized by acute onset of external ophthalmoplegia, ataxia, and altered consciousness, but may also present with limb weakness, pyramidal tract signs, long-tract sensory impairment, and autonomic dysfunction (5, 14). Autonomic dysfunction includes malignant arrhythmia and hemodynamic instability, which resemble the symptoms of TCM.

Recent evidence has shown that COVID-19 leads to anti-ganglioside antibody–mediated disorders such as GBS and Miller Fisher syndrome (2, 3). Regarding BBE, only one case report mentioned anti-GD1a antibody–positive atypical BBE occurring 1 month after SARS-CoV-2 infection, in which acute brainstem and cerebellar dysfunction developed without ophthalmoplegia (8). A 1-month interval seems too long to conclude that it was COVID-19–associated BBE because the mean latency between SARS-CoV-2 infection and presentation of anti-ganglioside antibody–mediated disorders has been reported to be 11 to 13 days (15). In contrast, our patient developed probable BBE according to the diagnostic criteria (5) at 2 weeks after SARS-CoV-2 infection and typically had autoantibodies against GQ1b and GT1a, plausibly suggesting that BBE was triggered by COVID-19.

The precise mechanism underlying the association between COVID-19 and anti-ganglioside antibody–mediated disorders is still unclear. One proposed mechanism is cross-reactivity between viral protein–associated gangliosides and peripheral nerve gangliosides as the result of molecular mimicry (4). However, Keddie et al. recently reported that SARS-CoV-2 proteins, including the spike protein, have no significant similarity to any known human proteins (16), which accounts for a strikingly lower rate of seropositivity for anti-ganglioside antibodies in COVID-19–associated GBS (7%) (15) than in conventional GBS (60%). Since our patient presented with BBE with anti-ganglioside autoantibodies 2 weeks after SARS-CoV-2 infection, the molecular mimicry theory seemed more plausible than the secondary epitope spreading theory for SARS-CoV-2 infection. Further studies are needed to clarify this issue.

Among autoimmune neurological disorders, GBS is well known to cause TCM. However, reports of TCM associated with BBE are rare. Only one previous report described a 62-year-old woman who developed TCM during the acute stage of BBE and required intubation and inotropic support due to decreased consciousness and severe hypotension (17). The precise pathophysiology of TCM is still controversial, but the most likely cause is catecholamine stress induced by a variety of physical and emotional stressors in patients with underlying disease or even in healthy individuals (18). Among the multiple etiologies for these stressors, direct involvement of the cardiovascular autonomic center in the medulla oblongata has been reported in central nervous system diseases such as multiple sclerosis (19). Thus, BBE might be associated with TCM via brainstem involvement, which influences excessive and sustained activation of the sympathetic nervous system. Nevertheless, it is possible that TCM has so far been underdiagnosed in patients with BBE due to masking of chest symptoms by altered consciousness or misdiagnosis as BBE-related cardiovascular autonomic dysfunction *per se* (20). Another important possibility is that COVID-19 is a trigger of TCM. Giustino et al. documented TCM in 4.2% of 118 consecutive patients with laboratory-confirmed COVID-19 (21). TCM occurs mostly during the acute phase of COVID-19 (11). In our patient, the presence of TCM was confirmed 2 weeks after the onset of COVID-19 and the timing of TCM improvement coincided with IVIG treatment for BBE (Figure 2), favoring BBE over COVID-19 as the cause of TCM. IVIG therapy might have exerted a marked favorable impact on TCM, presumably through anti-inflammatory actions on the cardiovascular autonomic center in the brainstem. However, simultaneous conventional TCM treatment might also have been effective.

In conclusion, in the setting of the COVID-19 pandemic, BBE might be triggered by COVID-19 as well as GBS. The possibility of TCM should be always considered because both BBE and COVID-19 are important risk factors for TCM.

## Data Availability Statement

The raw data supporting the conclusions of this article will be made available by the authors, without undue reservation.

## Author Contributions

MKi, SH, KTan, MH, KTak, YM, HJ, and HD examined and treated the patient. MKo assessed serum anti-ganglioside antibodies. HT and FT designed and supervised this study. MKi, SH, HT, and FT wrote the manuscript. All authors contributed to the article and approved the submitted version.

## Conflict of Interest

HT is an Associate Editor of *Frontiers in Neurology* and *Frontiers in Immunology*. The remaining authors declare that the research was conducted in the absence of any commercial or financial relationships that could be construed as a potential conflict of interest.

## Publisher's Note

All claims expressed in this article are solely those of the authors and do not necessarily represent those of their affiliated organizations, or those of the publisher, the editors and the reviewers. Any product that may be evaluated in this article, or claim that may be made by its manufacturer, is not guaranteed or endorsed by the publisher.
